# Editorial: Humanitarian Health in Conflict and Violence Settings

**DOI:** 10.3389/fpubh.2022.946090

**Published:** 2022-06-20

**Authors:** Claire J. Standley, Aisha Obad Jumaan, Erin M. Sorrell, Charbel El Bcheraoui

**Affiliations:** ^1^Center for Global Health Science and Security, Georgetown University Medical Center, Washington, DC, United States; ^2^Heidelberg Institute of Global Health, University of Heidelberg, Heidelberg, Germany; ^3^Independent Consultant, Yemen Relief and Reconstruction Foundation, Mercer Island, WA, United States; ^4^Department of Microbiology & Immunology, Georgetown University Medical Center, Washington, DC, United States; ^5^Center for International Health Protection, Robert Koch Institute, Berlin, Germany

**Keywords:** humanitarian health, conflict and violence, health equity, Yemen, Iraq, Afghanistan, Ethiopia, Lebanon

In late 2020, we launched a Research Topic on humanitarian health. We deliberately kept the scope broad to showcase diverse health topics and geography, especially from countries impacted by violence and conflict not commonly represented in scientific publications. These environments provide distinct and varied challenges for research and provision of health services compared to other types of humanitarian emergency. We aimed at assembling a collection of papers highlighting the extraordinary work performed by individuals and groups to both advance health, and, simultaneously, generate evidence that can be used to improve outcomes in similar settings and circumstances. The resulting submissions achieved our aim. Given that the COVID-19 pandemic was raging during the entire review period of the submissions, the issue includes several papers on COVID-19, albeit from diverse perspectives and populations. Here, we summarize the key findings and implications of the papers and include observations derived from this experience around conducting and publishing health research in conflict and violence-affected humanitarian settings.

In total, six papers were accepted to the Research Topic, representing substantial geographical diversity in conflict settings: Yemen, Iraq, Afghanistan, Lebanon, and Ethiopia. Most of the papers sought to examine the influence of conflict on health. For example, Dureab et al. analyzed the ways in which conflict has influenced fragmentation of the Yemeni health system. The authors identify different forms of fragmentation, and note that while exacerbated by conflict, the fragmentation of Yemen's health system is multifactorial and existed prior to the current period of violence. Prioritizing support and capacity strengthening for local and national health authorities is identified as a key opportunity for successfully implementing programs, especially related to integration of primary care.

Focusing on the health workforce, Al Serouri et al. described the Yemen Field Epidemiology Training Programs (FETP)'s role in responding to the COVID-19 pandemic in a conflict setting. The Yemen FETP used WHO's nine pillars of COVID-19 preparedness and response. The paper concluded with two main lessons relevant to Yemen and other war-torn countries: 1) as access to outside experts becomes limited in conflict times, it is crucial to invest in building national expertise to provide timely, cost-effective and sustainable services, and 2) to overcome response delays, it is essential to also build such expertise at the governorate and district levels because they are the first respondents.

Indeed, concentrating efforts at the national level in conflict-afflicted settings often does not address the problem appropriately. Geographic variations and disparities are well highlighted by Comfort et al. while describing vaccine coverage and incident cases of measles in Iraq between 2001 and 2016. The authors further shed light on a worse problem. Effective, and approved vaccines against measles have been long available. Still, in long-term conflict settings like Iraq, even vaccine campaigns cannot control measles' incidence when vaccine coverage is hindered due to population displacement.

Parray et al. addressed the challenges of the health workforce by analyzing the motivation, status within the health system, and difficulties faced by female community health care workers (CHWs) in Afghanistan as they provide essential services in a difficult environment and a conflict setting. Although CHWs view their work as essential, several socio-cultural and political obstacles are identified. These include lack of recognition and compensation or delayed payment for their work, lack of acknowledgment, reward for good performance and progression in their careers. The authors conclude CHWs should be supported and acknowledged by the national healthcare system as being experts in their field, and be provided proper training and accreditation, opportunities for career promotion, and included in the planning and decision making of the healthcare program.

Messages to raise awareness on infection prevention must consider the local context and address an ever-increasing “knowledge-practice” gap. Tsegaye et al. qualitative work outlines the disconnect between awareness raising and behavioral change for COVID-19 mitigation and prevention measures in refugee camps. The authors found that for members of the Nguenyyiel refugee camp in Gambella, Ethiopia knowledge of a risk did not guarantee uptake of preventative measures. As with other refugee settings, significant structural and cultural challenges affected behavior at the individual, household and community level impacting the application of various measures. Comprehensive strategies must consider delivery mechanisms, misconceptions and cultural factors as well as incentives to preventive behaviors. These strategies when applied correctly may even be effective in addressing other health priorities across the camp.

Finally, Abouzeid et al. perspective article summarizes the impacts of Lebanon's complex humanitarian crisis on children's health in reversing previous modest gains toward achieving development goals. The paper includes numerous, detailed recommendations to improve conditions for children and their families, including calling for a locally-led and needs-based humanitarian response; building the evidence-base to inform action and drive accountability; and mobilizing the Lebanese diaspora, all of which are underscored by the imperative for political reform.

To this end, despite the diversity of settings, we see a number of common themes emerging. Perhaps most central among them is the importance of providing support and capacity strengthening for local experts, authorities, and organizations, and critically, allowing these local actors autonomy and agency over how to prioritize and implement response actions.

It is also worth noting that in addition to the geographical diversity of the papers' subject matter, the authors themselves are based in institutions from 12 countries: Yemen, USA, Italy, Bangladesh, Lebanon, Germany, Ethiopia, Kenya, United Kingdom, Australia, India and Iraq, in descending order of frequency (Figure 1). Approximately one third of the authors (13/35) were based solely in a conflict-affected country.

**Figure 1:**
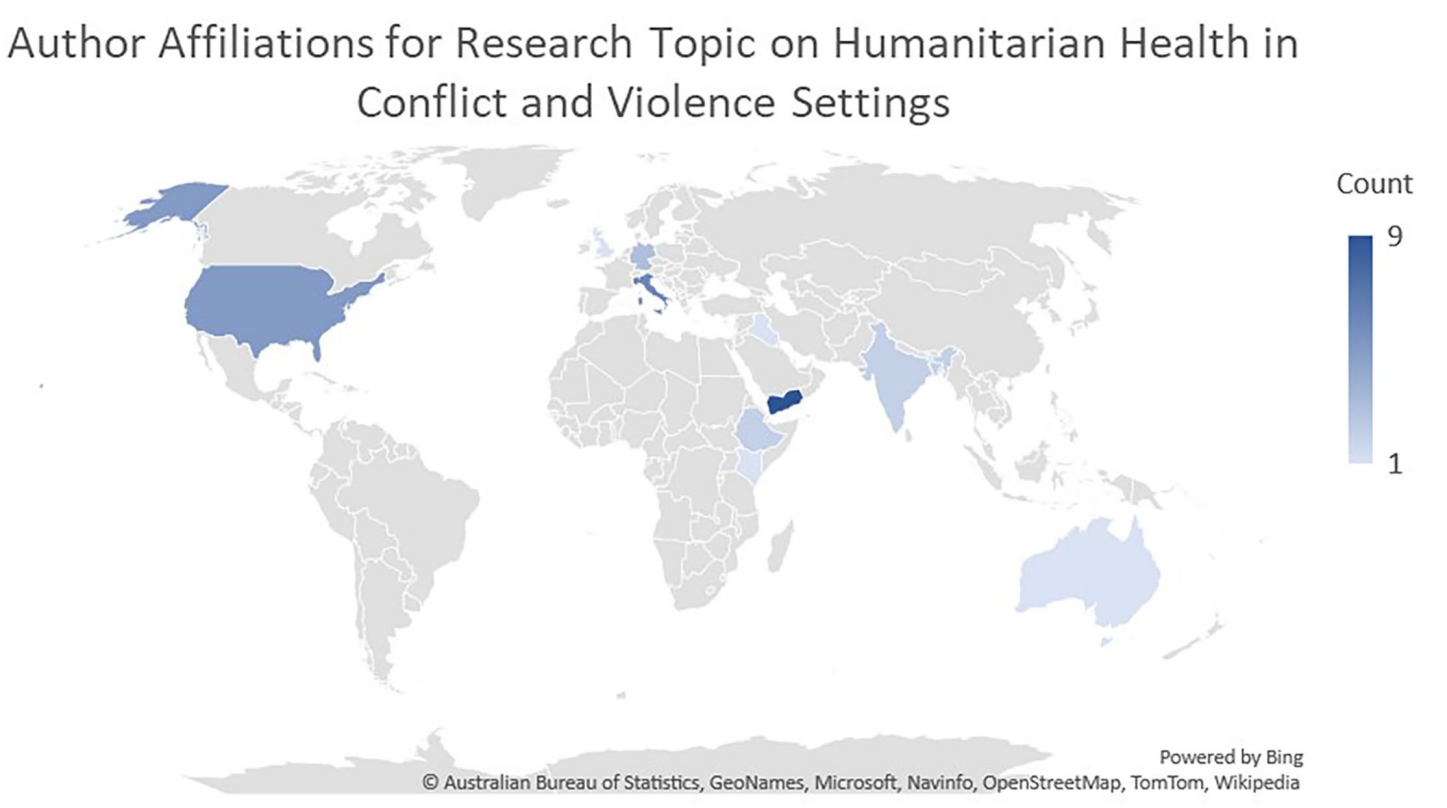
Map showing the country affiliations of authors contributing to the Research Topic. The darkness of the color represents the number of authors listing an affiliation in that country.

This geographical diversity of authors demonstrates substantial international collaboration in humanitarian health topics. However, the relatively low proportion of authors based solely in conflict-affected countries suggests that it can be challenging to ensure participation of individuals working and/or living in these settings as co-authors on research publications. Further, we observed directly as guest editors for the Research Topic that language and financial barriers were at play.

Authorship can be a challenging issue when it comes to international collaborations in general, and perhaps particularly so in conflict- or violence-affected humanitarian settings. The funding for research projects is often managed by international partners, who regulate how findings are disseminated. International partners from academic institutions may be particularly motivated to publish their research in scientific journals, and take the lead in drafting manuscripts. Conversely, local partners may more likely be involved in field work, including the substantial logistical, security, political and coordination aspects required to implement the work. This division of labor may also impact authorship position, with international partners taking the more prestigious first and last positions, at the expense of local collaborators (1).

This series of papers also reflected the diversity of sectors and institutions involved in humanitarian health research, with authors representing governmental institutions, non-governmental organizations, independent experts and academia. This highlights the importance of journals, and peer-reviewers, in recognizing the value of expertise separately from institutions for humanitarian health research.

Regardless of the sector, the cost of publication can be a barrier to research dissemination for authors based in middle-income countries in general, but more specifically those affected by conflict or violence. Fee waivers are often not available, or only cover a fraction of the cost, despite substantial challenges in accessing and transferring large sums of money in these settings. For example, Lebanon an upper-middle income country in severe economic crisis lacks access to foreign currency, specifically in USD, making it almost impossible to transfer money internationally. This restriction severely limited the ability of one of our topic's corresponding authors to pay the article processing fees. In this case, the journal was able to offer a full waiver, but this type of exception may not always be available. The effort required to apply for fee waivers, and the risk that one might not be granted, may limit the willingness of authors from conflict-affected countries to serve as corresponding authors.

To conclude, the papers in this Research Topic provide new evidence-based data on health programs in conflict and violence-affected humanitarian settings while providing insights into some of the challenges and opportunities around publishing this research. We must acknowledge the multi-sectoral nature of humanitarian health research, and incentivize publication support and structures accordingly to meet the needs and priorities of diverse contributors. We need to address funding constraints, especially around open access fees and publication fee waivers. As a scientific community, we must address equity around representation of local contributors as co-authors, especially recognizing non-writing roles. Similarly, we should find ways to support inclusion of non-traditional voices in academic publications, including unaffiliated experts, government officials, and community members, through a stronger emphasis on co-production and co-dissemination. This would allow the body of scholarship on humanitarian health topics to reflect its global reality.

## Author Contributions

CS wrote the first draft of the editorial. CS, AJ, ES, and CE each contributed summaries of Research Topic papers, reviewed drafts, and provided edits. All authors approved the final version.

## Conflict of Interest

The authors declare that the research was conducted in the absence of any commercial or financial relationships that could be construed as a potential conflict of interest.

## Publisher's Note

All claims expressed in this article are solely those of the authors and do not necessarily represent those of their affiliated organizations, or those of the publisher, the editors and the reviewers. Any product that may be evaluated in this article, or claim that may be made by its manufacturer, is not guaranteed or endorsed by the publisher.
